# Health and socioeconomic resource provision for older people in South Asian countries: Bangladesh, India, Nepal, Pakistan and Sri Lanka evidence from NEESAMA

**DOI:** 10.1080/16549716.2022.2110198

**Published:** 2022-12-20

**Authors:** Natasha Roya Matthews, George James Porter, Mathew Varghese, Nidesh Sapkota, Murad Moosa Khan, Ammu Lukose, Stella-Maria Paddick, Malathie Dissanayake, Naila Zaman Khan, Richard Walker

**Affiliations:** a Faculty of Medical Sciences, Population Health Sciences Institute, Newcastle University, Newcastle upon Tyne, UK; b Department of Psychiatry, National Institute of Mental Health and Neuro-Sciences (NIMHANS), Bengaluru, India; c Department of Psychiatry, B.P. Koirala Institute of Health Sciences, Dharan, Nepal; d Brain and Mind Institute, Aga Khan University, Karachi, Pakistan; e Centre for Community Mental Health (CCMH), Mangalore, India; f Department of Psychology and Counselling, The Open University of Sri Lanka, Nawala, Sri Lanka; g Clinical Neurosciences Center, Bangladesh Protibondhi Foundation, Dhaka, Bangladesh

**Keywords:** Ageing, elderly health, mental health, social care, South Asia

## Abstract

**Background:**

The global population is ageing rapidly, with low- and middle-income countries (LMICs) undergoing a fast demographic transition. As the number of older adults in LMICs increases, services able to effectively address their physical and mental health needs will be increasingly important.

**Objective:**

We review the health and socioeconomic resources currently available for older people in South Asian countries, Bangladesh, India, Nepal, Pakistan and Sri Lanka, to identify gaps in available resources and assess areas for improvement.

**Methods:**

We conducted a search of grey and published literature via Google Search, Compendex, EBSCO, JSTOR, Medline, Ovid, ProQuest databases, Scopus and Web of Science. Data on population demographics, human resources, health funding and social protection for older people were extracted. Local informants were consulted to supplement and verify the data.

**Results:**

In the study countries, the number of health professionals with expertise in elderly care was largely unknown, with minimal postgraduate training programmes available in elderly medicine or psychiatry. Older adults are therefore cared for by general physicians, nurses and community health workers, all of whom are present in insufficient numbers per capita. Total average healthcare expenditure was 2.5–5.5% of GDP, with 48.1–72.0% of healthcare costs covered by out-of-pocket payments. Pakistan did not have a social pension; only India and Nepal offered financial assistance to people with dementia; and all countries had disproportionately low numbers of care elderly homes.

**Conclusions:**

Inadequate healthcare funding, a shortage of healthcare professionals and insufficient government pension and social security schemes are significant barriers to achieving universal health coverage in LMICs. Governing bodies must expand training programmes for healthcare providers for older adults, alongside increasing social protection to improve access to those in need and to prevent catastrophic health expenditure.

## Background

The global population is ageing rapidly, with the number of those aged 60 years or over predicted to rise from 962 million in 2017 to 2.1 billion in 2050 and 3.1 billion in 2100 [1]. Most older people live in low- and middle-income countries (LMICs), with the largest number in Asia. This older population is rapidly expanding due to the continuing demographic transition [2]. At the same time, these older populations have experienced a massive increase in the prevalence of non-communicable diseases (NCDs) [3], and the syndrome of frailty has been increasingly recognised [4]. Currently, services are not well adapted for providing care for older people with long-term conditions, particularly those with significant comorbidity [5].

Multimorbidity increases significantly with age and is common in LMICs (mean standard prevalence of 7.8%) [6]. A population-based cross-sectional survey of LMICs highlighted that multimorbidity is strongly associated with adverse effects on older adults' quality of life, physical functioning and mental health [7]. Similarly, a large population-based cohort study of frailty indicators in LMICs identified an association between frailty (including cognitive impairment) and dependence and mortality in older people [8]. Hence, it is necessary to provide services to address the interdependent physical and mental health needs of chronically multimorbid and frail older people in LMICs. Approaches focused solely on a single disease process or psychiatric disorder, while excluding socioeconomic factors, comorbidities and disabilities in older people, are unlikely to be successful or sustainable.

With the United Nations Sustainable Development Goal (SDG) 3, there is a growing trend for universal healthcare coverage (UHC) within LMICs, particularly for vulnerable groups such as older adults. Moreover, the World Health Organization (WHO) has recognised that ‘without considering the health and social care needs of the ever-increasing numbers of older people, SDG 3 will be impossible to achieve’ [9]. The associated healthcare costs may be covered by insurance schemes, taxation, out-of-pocket payments or a combination of two or more of these. Countries have decided to tackle these issues in different ways, both in terms of direct legislation and guidelines for practise in healthcare and social care sectors. Lessons can be learned from understanding the systems in different countries, and how well different ideas work in practice.

Mental and substance use disorders account for 7.4% of all-age disease burden worldwide, and their contribution to the global burden of disease is rising [10]. Stressors such as bereavement, inadequate social support and isolation are prevalent in older adults and contribute to worsening mental health. A systematic review of the prevalence of common mental disorders found that 29.2% of adults (aged 16–65) experience mental and substance use disorders in their lifetime, with a disproportionate number in LMIC settings [11]. Worldwide, an estimated 322 million people (of all age groups) live with depression and 264 million with anxiety [12]. Specific prevalence figures for older adults are not available, reflecting the suboptimal screening and identification of these disorders in older adults. Given the data available on the general adult population, it is likely that the older adult population also experiences significant disease burden from mental and substance use disorders.

Despite the substantial burden of mental health disorders worldwide, the gap between mental health service needs and provision in LMICs persists [13–15]. The WHO states that resources available are ‘insufficient, inequitably distributed, and inefficiently used, which leads to a treatment gap of more than 75% in many countries with low and middle incomes’ and increasingly recommends scaling up mental healthcare, through integration into primary healthcare and general medical services [13]. Specifically, this could be achieved by training non-specialist primary care workers to diagnose and treat mental disorders [13,16,17]. Similarly, despite the need for an accessible primary health service, adapted with trained workers equipped to diagnose and treat the needs of older people, likely to have multiple comorbidities [18,19], the gap in provision of healthcare services specifically for older people is not well characterised.

There is an increasing awareness of the contribution of poverty, social and environmental factors to mental disorders and disability in older people worldwide, particularly in LMICs [20]. Inequities in healthcare access for older people are well recognised in LMICs: a recent population-based cross-sectional survey of 17,994 individuals aged 65 years and over noted a positive correlation between higher education, more household assets, receiving a pension, health insurance and the proportion using healthcare services [21].

The North East England South Asia Mental Health Alliance (NEESAMA) inaugural meeting took place on the third and fourth of November 2018 in Kathmandu, Nepal. It was attended by clinician representatives from each country (psychiatrists, psychologists and other health professionals) working in older adults’ services. Further contacts were identified to establish a full clinical and academic network focused on improving older people’s mental health services in South Asian countries. It was agreed among delegates that the biggest concern, regarding the care of older adults, is related to the provision and cost of medical and social care for older people. It was also recognised that very few data are published outlining the current provision. Thus, the need for data from South Asian countries concerning socioeconomic resource provision for older people was established, and a plan of action was formalised.

We, therefore, sought to gather and review data from Bangladesh, India, Nepal, Pakistan and Sri Lanka, about provision for older people to identify gaps in health and socioeconomic provision, as well as to identify examples of good practice, which may impact the physical mental and social wellbeing of older people in South Asia.

## Methods

Data were collated from a search of English-language grey and published literature through the search engine Google Search and Newcastle University Library Search (including major databases Compendex, EBSCO, JSTOR, Medline, Ovid, ProQuest databases, Scopus and Web of Science), using relevant keywords. Where specific data were not available online, we supplemented the search by contacting specific country governing bodies and informants to identify all the relevant data from the study countries concerning provision for older people.

Information on health and social care provision for older adults in all six countries focused on the following categories.

- Population demographics
  - Population size
  - Population aged 60 and over
  - Life expectancy
  - Gross Domestic Product (GDP)
- Human resources
  - Doctors: geriatricians, primary care physicians, psychiatrists and old-age psychiatrists
  - Nurses or midwives
  - Specialist nurses
  - Physiotherapists
  - Occupational therapists
  - Speech and language therapists
- Health funding
  - Taxation/Government
  - Insurance scheme/Pre-paid
  - Out-of-pocket
  - Development assistance
  - Total
- Social protection
  - Retirement age
  - Old-age social pension
  - Special dementia social protection
  - State care home provision

NRM carried out a subsequent round of data collection to check for accuracy and expand the initial data set. Summarised data were finally checked for accuracy by the NEESAMA team delegates who provided additional data sources to address relevant gaps. Descriptive statistics were calculated for quantitative analysis of numeric data. Non-numeric data were analysed by identifying and summarising key themes.

## Results

### Population demographics

Table 1 summarises the population demographics of the study countries, as stated by the World Bank. Except for India, all study countries have populations growing, at or above the global average rate of 1.1%, with Pakistan growing at a significantly higher rate than the other countries at 2.1% [22]. Apart from Sri Lanka, the percentage of the population aged 65 and above is around 4–6%, significantly below the global average of 8.9%, yet this is increasing quickly in all study countries [22]. The study countries’ life expectancy is generally close to the worldwide average life expectancy at birth of 72 years, but lower than that of high-income countries. The range between the highest (Sri Lanka) and the lowest (Pakistan) is 10 years. The significant variation in the population characteristics across the study countries indicates variation in the level of resources required for older persons’ health. Meanwhile, the wide range in GDP per capita means that the resources available to meet those requirements are significantly different in each country.

**Table 1.** Population demographics, 2018 [22].

	Bangladesh	India	Nepal	Pakistan	Sri Lanka
Population, total	161,356,039	1.353 billion	28,087,871	212,215,030	21,670,000
Population growth (annual %)	1.050	1.037	1.654	2.056	1.048
Population aged 65 and above, total	8,323,375	83,591,151	1,608,781	9,152,355	2,269,547
Population aged 65 and above (% of total population)	5.158	6.18	5.728	4.313	10.473
Life expectancy at birth, total (years)	72.052 (2017)	69.165 (2017)	70.169 (2017)	66.947 (2017)	76.648 (2017)
Literacy rate, adult total (% of people ages 15 and above)	73.912	74.373	67.908	59.132 (2017)	91.71
GDP (current US$)	274.025 billion	2.719 trillion	29.04 billion	314.588 billion	88.901 billion
GDP per capita (current US$)	1,698.263	2,009.979	1,033.912	1,482.403	4,102.481
Poverty headcount ratio at $1.90 a day (2011 PPP) (% of population)	14.8 (2016)	21.2 (2011)	15.0 (2010)	3.9 (2015)	0.8 (2016)
Current health expenditure (% of GDP)	2.365 (2016)	3.658 (2016)	6.294 (2016)	2.753 (2016)	3.893 (2016)

### Human resources

Table 2 shows the publicly available data on the numbers of different healthcare professionals potentially involved in older persons’ health; however, some will also work with other age-groups. The number of geriatricians in many of the study countries is not known. In fact, in countries such as India [23,24] and Pakistan [25], it is well documented that geriatrics is not yet an established speciality training pathway, and general physicians or primary care doctors usually provide care of older adults. In instances where country level statistics were not published, estimates of human resources were provided via email from field experts, including N Sapkota from Pakistan in 2019, MM Khan from Pakistan in 2020, and The Association of Nepal's Occupational Therapists in 2019 (unreferenced).

**Table 2.** Human resources for older persons’ health.

	Bangladesh	India	Nepal	Pakistan	Sri Lanka
Physicians (per 1,000 people) [22]	0.472 (2015)	0.758 (2016)	0.598 (2014)	0.978 (2015)	0.881 (2015)
Geriatricians	Not available	Not available	1^1^	5–10^2^	3 [63]
Psychiatrists (per 100,000 people)	0.130 (2016) [26]	0.292 (2016) [26]	0.356 (2016) [26]	0.185 (2011) [60]	0.517 (2017) [26]
Old-age psychiatrists	0 (2017) [27]	Indian Association of Geriatric Mental Health members: 583 [28]	1 (2019)^1^	5–10 (2020)^2^	Not available
Nurses and midwives (per 1,000 people) [22]	0.267 (2015)	2.094 (2016)	2.041 (2014)	0.502 (2015)	2.794 (2015)
Nurses working in mental health sector (per 100,000 population)	0.873 (2016) [26]	0.796 (2016) [26]	0.558 (2016) [26]	7.384 (2011) [60]	3.278 (2017) [26]
Social workers working in mental health sector (per 100,000 population)	Not available	0.065 (2016) [26]	Not available	1.702 (2011) [60]	0.285 (2017) [26]
Psychologists working in mental health sector (per 100,000 population)	0124 (2016) [26]	0.069 (2016) [26]	0.523 (2016) [26]	0.259 (2011) [60]	0.246 (2017) [26]
Community health workers (per 1,000 people) [22]	0.476 (2012)	0.581 (2016)	0.684 (2004)	0.087 (2015)	Not available
Physical therapists	2,135 (2019) [29]	Indian Association of Physiotherapists members: >50,000, includes physiotherapists, and physiotherapist students undergoing the graduation and PG courses from recognised physiotherapy colleges [30]	402 (2019) [31]	15,000 (2017) [32]	360 (2020) [33]
Occupational therapists	Bangladesh Occupational Therapy Association members: 280 [34,35]	5000 (2015) [78]	10 (2019) – all members trained abroad^3^	~19,000, includes occupational therapists, physical therapists, speech-language pathologists, psychologists, medical interns and dietitians [36]	Not available
Speech and Language therapists	Not available	Indian Speech and Hearing Association members: >3440, includes speech-language pathologists, audiologists, and speech, language, and hearing scientists [37]	20–25 audiologists who practise speech therapy^1^	7 [38]	100 [39]

From 2014 to 2016, the ratio of psychiatrists in the study countries was significantly lower than that in high-income countries, which comparatively employ 12 psychiatrists per 100,000 people [40]. Limited data were available in terms of old-age psychiatrists, suggesting it is unlikely this exists as a sub-speciality. India is the exception, with two institutes offering dedicated training in old-age psychiatry: King George’s Medical University (KGMU) and the National Institute of Mental Health and Neuro-Sciences (NIMHANS) [41]. Most of these countries have specialist mental health nurses or community health workers, however, in low numbers per capita. In countries such as Bangladesh and India, speech and language therapists (SALTs) cover a broad range of services in various settings that include services for older adults [42,43].

#### Summary of country reports on the status of geriatrics and old-age psychiatry

##### Bangladesh

The directory of doctors does not list geriatrics as a speciality [44], implying that specialist geriatricians are, at best, rare. Barikdar et al. [45] highlight how taking care of older adults will be a significant challenge for Bangladesh due to inadequate resources allocated to services for older adults and no proper planning or strategic interventions for providing holistic care. Indeed, other than the Dhaka Medical College Hospital's special geriatric unit opening in April 2014, there are no other public-sector activities [46,47]. There is, however, ongoing work by the Bangladesh Association for the Aged and Institute of Geriatric Medicine (BAAIGM), a non-governmental organisation (NGO) operating specific healthcare and rehabilitation programmes for older people. The BAAIGM acts nationally as an advocate for improved geriatric care and researches the health and socioeconomic status of older adults in Bangladesh [48,49]. The BAAIGM also runs a 50-bed geriatric hospital, 50-bed dormitory for older adults and is constructing a rehabilitation centre in Gazipur with a capacity for up to 500 older adults [47]. Despite the lack of public-sector geriatric care, private institutions such as the Subarta Trust and Sir William Beveridge Foundation work to improve welfare for older adults. The Subarta Trust operates a residential complex with geriatricians and allied healthcare professionals, although such services are unaffordable to the urban middle class and poor [47,50]. The William Beveridge Foundation provides home care services to around 150 vulnerable older people and access to trained geriatric doctors and physiotherapists [47,51].

##### India

Postgraduate training programmes in geriatrics do exist, although few. Evans et al. [52] reported only that Madras Medical College has a full-time geriatric Doctor of Medicine (MD) programme, and Indira Gandhi National Open University runs a part-time Postgraduate Diploma in Geriatric Medicine. Currently, the Medical Council of India lists eight medical colleges offering a specific MD in Geriatrics, with a total annual intake (seats) of 42 [41]. The 2010 government policy effort – the National Programme for the Health Care of the Elderly (NPHCE) – was expected to produce a Regional Geriatric Center (RGC) in eight regional medical institutions, with a dedicated geriatric outpatient department and 30-bed geriatric ward, as well as trained geriatric healthcare workers, including postgraduates in geriatric medicine [53]. These government-funded facilities may be free or highly subsidised for all individuals aged over 60 [54]. However, it was not possible to ascertain the extent to which these changes have been successfully implemented, despite examination of available literature and consultation with local experts.

Moreover, the NHPCE has been criticised for its focus on care in institutions for older adults and neglecting preventative home-based measures, as well as failing to outline a decentralised vision that addresses regional differences [53]. Despite these discrepancies, Indian doctors have founded both the Indian Academy of Geriatrics [55] and Geriatric Society of India [56] dedicated to sharing knowledge and delivering improved care to older adults, the former of which has reportedly around 950 members, although not necessarily with specific qualifications in geriatrics. There has also been an influx of Indian geriatricians who previously worked overseas in the private sector (2020 email from M Varghese to NRM; unreferenced).

Following a decade of advocacy, in 2010, the Department of Geriatric Mental Health at KGMU was recognised by the Medical Council of India as a subspeciality academic department with an approved speciality training programme available to one candidate [57]. There are now two centres offering the DM (superspeciality course) in Geriatric Mental Health (KGMU) and Geriatric Psychiatry (NIMHANS), with three and two seats available to applicants, respectively, [41]. NIMHANS also offers one place on the Postdoctoral Fellowship (PDF) in Geriatric Psychiatry per year (2020 email from M Varghese to NRM; unreferenced).

##### Nepal

There is no specific training pathway for geriatricians; however, the Nepalese Society for Gerontology and Geriatrics (NSGG) is an established NGO, working to further the agenda for care of older adults and to promote geriatric research and training [58]. Currently, NSGG is working with the government to design a training pathway for geriatric nursing and partnering with other agencies to train General Practitioners in identifying and managing geriatric syndromes (2019 email from the Nepalese Society for Gerontology and Geriatrics to GJP; unreferenced). Shrestha [59] outlined how Nepal’s Government adopted a national policy, legislation and regulations on ageing, including setting up geriatric wards in selected regional hospitals. However, limited resources present an ongoing challenge to implementing programmes for the welfare of older adults [59]. Additionally, information on the specific number and development of government hospitals and medical colleges with specialist geriatric units could not be found.

##### Pakistan

Geriatrics is not yet an independent speciality with a respective training programme. Older patients are treated by general medical practitioners and primary care physicians without access to specialist services for older adults, such as mental health services, or rehabilitation centres for fractures, stroke or movement disorders. In 1999, the government put forth a National Policy to promote better health of older people, with plans to train a group of healthcare providers for older adults, including primary care doctors in geriatrics, physical therapists and social workers; however, this has not been implemented [25]. Recent data regarding human resources for health in Pakistan were particularly sparse. Anecdotally, Professor of the Department of Psychiatry at Aga Khan University, M. Khan (2020 email from M Khan to NRM; unreferenced), reported no more than 5–10 geriatricians in the country, and only 5–10 clinicians with higher training in old-age psychiatry (having completed either a US Fellowship or the UK speciality training programme abroad). Of 520 psychiatrists [60], almost all are general adult psychiatrists, with limited facilities for sub-specialities such as old-age psychiatry, child and adolescent psychiatry, forensic psychiatry and substance abuse. Therefore, mental disorders of older adults, such as depression and dementia, are managed variously by general adult psychiatrists, neurologists or general medical specialists. Similarly, there is no separate curriculum or training in mental health for nurses. General nurses may opt to work in a psychiatric setting and subsequently ‘learn on the job’ (2020 email from M Khan to NRM; unreferenced).

##### Sri Lanka

Historically, care for older adults is delivered by general physicians on general medical wards and through specialist health services, including mental health, disability and rehabilitation, though such services are not explicitly aimed at older adults. In 2013, however, the University of Colombo Postgraduate Institute of Medicine (PGIM) pioneered the first ‘speciality training’ Postgraduate Diploma in Geriatric Medicine, supported by the Ministry of Health authorising successful applicants one-year release from their posts [61]. Since 2017, the PGIM also offers an MD in Geriatric Medicine [62], with two seats available (2020 email from M Dayabandara to MD; unreferenced). However, the Sri Lankan directory of doctors currently lists only three geriatric physicians [63]. Such programmes aim to train medical professionals to provide care to older adults in a diverse range of settings (such as hospitals, residential care facilities and the community), promote positive attitudes towards caring for older adults and ensure active, healthy ageing in Sri Lanka. Following the Protection of the Rights of Elders Act in 2000, the government established a National Council for Elders, with representatives from government ministries, the voluntary sector and experts, to develop and implement programmes to protect and promote the rights of elders. Such activities include funding access to psychological counselling, day centres and home-care, as well as trained carers for older adults [61]. There are also several initiatives in progress to improve government health service provision for older adults, including older person-friendly hospital wards, health clinics, a stroke unit in each district general hospital and a stroke centre in each province [61]. In 2014, Sri Lankan doctors also launched the Sri Lanka Association of Geriatric Medicine to promote geriatric education and research among the medical profession and public, by facilitating ‘elderly-friendly environments’ in healthcare institutions and communities and coordinating the work of different organisations promoting the welfare of older adults [64]. Despite the notable increase in health policies and initiatives related to older adults in recent years, a lack of organisation and integration of existing health infrastructure and systems persists. Furthermore, the actual progress of government initiatives remains unclear.

The National Institute of Mental Health is the largest mental health hospital in Sri Lanka, with a specific Psycho-Geriatric Unit to treat older adults, particularly dementia [65]. However, none of the team of consultants have listed old-age psychiatry credentials [66]. In 2017, PGIM also commenced an MD in Old-Age Psychiatry and had since accredited several training centres to deliver this course; four trainees are undergoing this training, and the first old-age psychiatrist is expected to be Board Certified in 2022 (2020 email from M Dayabandara to MD; unreferenced).

### Health funding

Table 3 shows the average healthcare spending by type of financing and total health expenditure per person in 2017. Nepal’s health spending as a percentage of GDP was significantly greater than that of other countries; however, all government healthcare expenditures were below the global average of 10.0% and less than the LMIC average of 5.3% [22], except for Nepal. Furthermore, government spending in all study countries, except Sri Lanka, was less than the WHO spending target for LMICs of US$ 60 per capita by 2015 to deliver essential health interventions [67].

**Table 3.** Average healthcare expenditure, 2017 [68].

	Bangladesh	India	Nepal	Pakistan	Sri Lanka
Government ($ per person)	7.24 (5.50–9.32)	18.25 (13.35–23.78)	11.21 (8.44–14.34)	10.41 (7.81–13.52)	64.74 (50.91–81.63)
Pre-paid private ($ per person)	1.18 (0.56–2.26)	6.88 (3.35–12.39)	4.60 (2.11–8.68)	1.76 (0.87–3.15)	9.27 (4.68–17.40)
Out-of-pocket ($ per person)	29.22 (20.94–40.33)	43.20 (32.03–60.02)	30.59 (22.14–41.48)	22.15 (15.49–30.71)	72.92 (55.37–95.34)
Development assistance for health ($ per person)	2.72	0.60	3.65	2.74	4.65
All-cause total ($ per person)	40.36 (31.49–51.66)	68.93 (54.44–87.48)	50.06 (39.51–63.09)	37.06 (29.22–46.28)	151.59 (124.15–182.03)
All-cause total (% of GDP)	2.48 ((1.92–3.21)	3.53 (2.80–4.49)	5.49 (4.29–7.02)	2.81 (2.19–3.51)	3.95 (3.24–4.77)

**Figure 1.**
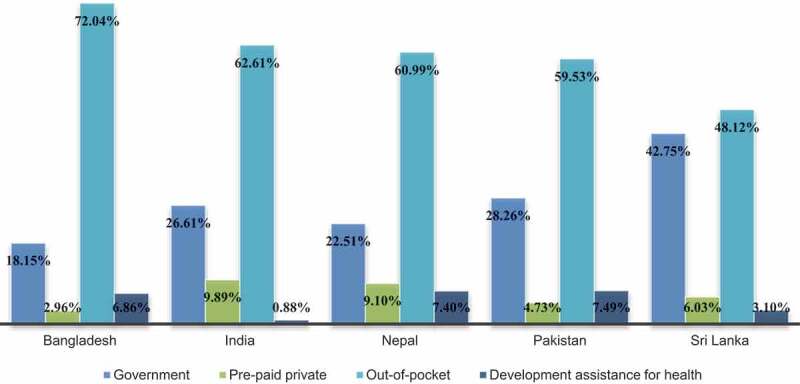
Type of healthcare financing, as % total healthcare expenditure, 2017 [68].

Figure 1 shows the different types of healthcare financing as a proportion of average total healthcare expenditure. In all the study countries, healthcare costs were primarily covered by out-of-pocket spending with between 48.1% and 72.0% of costs accounted for by out-of-pocket payments. Government-financed healthcare expenditure was low at only 18.2–28.3% in Bangladesh, India, Nepal and Pakistan. Sri Lanka stands out as the exception, with government spending covering 42.8% of healthcare costs and the smallest proportion out-of-pocket payments.

### Social protection

Most of the study countries have established some social protection for older adults in an old-age social pension. However, the Government of Pakistan operates a social insurance system, providing contributory pensions to retired employees and reportedly reaching just 2.30% of people aged over 65 [69]. For the purpose of this research, we focus on a social (or non-contributory) pension paid by the state to an individual upon their retirement. According to the World Bank's five-tier pillar framework, social pensions are known as Pillar zero ‘non-contributory social assistance financed by the state, fiscal conditions permitting’ [70].

Table 4 shows old-age social pension adequacy in Bangladesh, India, Nepal and Sri Lanka. The percentage of GDP allocated to such social pensions is low, and apart from in Nepal, the monthly benefit is significantly below the poverty line of $1.90 per day, as defined by the World Bank. The combination of a very low, or in Pakistan’s case, non-existent; social pension with high out-of-pocket payments for healthcare poses a significant barrier to accessing adequate healthcare, especially for the poorest and most vulnerable older adults.

**Table 4.** Old-age social pension adequacy [71].

	Bangladesh	India	Nepal	Sri Lanka
Monthly benefit (local currency)	500 takas	200 rupees	2,000 rupees	2,000 rupees
Monthly benefit ($)	6	3	19	13
% of older people (65+) receiving social pension	34.90	17.74	46.80	No data
% of population over age of eligibility covered	34.9	17.7	79.9	32.8
% of GDP per capita	5.2	2.2	30.8	3.8
% of $1.90 poverty line (PPP)	29	20	110	72
Cost of social pension as % of GDP	0.102	0.035	0.703	No data

Appendix A details the national retirement age for each study country and the social protection programmes available to older adults, including special provision for those with dementia. In countries such as Sri Lanka, the retirement age of 55 is significantly lower than the Organisation for Economic Co-operation and Development (OECD) average of 64.3 [72], whilst the life expectancy of 76.6 (Table 1) is relatively close to the OECD-36 average of 80.9 [73]. This combination will inevitably lead to a growing elderly retired population unless there is a future change in retirement policy. Financial protection for those diagnosed with dementia was not available in most countries, apart from India and Nepal, the latter of which involved a contribution towards the cost of care at only four specific hospitals. Traditionally, care for older adults relied upon older persons co-residing with the family and receiving care at home; however, most study countries now have a limited number of state and privately funded care homes for older adults requiring long-term care.

## Discussion

### Principal findings

Bangladesh, India, Nepal, Pakistan and Sri Lanka have limited resources dedicated to older adults’ care, specifically human resources, health funding and social protection. Furthermore, publicly available data were limited, particularly regarding human resources for older persons’ health, especially mental healthcare. This finding is consistent with the well-documented gap in mental health treatment, prevention and quality of care [74].

A lack of geriatrics and old-age psychiatry as established specialities presents significant challenges to healthcare for older adults. Without specialist clinicians with a comprehensive knowledge of the health needs of older adults, health services may not be appropriately equipped to provide high-quality care. Although most study countries had government and non-governmental organisations working to promote and expand services for older adults, including geriatric health education, only India and Sri Lanka had established academic institutions offering postgraduate training programmes in geriatrics. This lack of regimented and established training programmes in a majority of the countries highlights the extent to which geriatric medicine has been overlooked. While India and Sri Lanka both offer a Postgraduate Diploma or MD in Geriatric Medicine, the number of potential seats is miniscule compared to the seats available for other medical or surgical specialities. Since 2011, only India offers postgraduate training in old-age psychiatry, with five available seats for the DM in Geriatric Psychiatry. Lodha and De Sousa [75] highlight the gross inadequacy of these numbers, reporting that India has 5000 general psychiatrists to cater for 21 million older persons with a reported need for mental health services. Furthermore, only an estimated 100 memory clinics provide diagnostic services, medication and post-diagnostic support to a population of over 100 million older adults [76].

Similarly, numbers of allied healthcare professionals per capita are very low (Table 2), indicating a range of services such as carers, physiotherapy, occupational therapy and speech and language therapy have insufficient staff to meet the growing requirements of the ageing population. The Comprehensive Geriatric Assessment, generally considered the gold standard assessment for multimorbid, frail older people, specifically outlines the need for a multidisciplinary approach [77]. Nevertheless, while most of the study countries have some access to a range of allied health workers, the number and training level were variable. For example, there are centrally accredited Masters and PhD-level programmes in most universities in India to train occupational therapists, ensuring that all those trained have a standardised skillset [78]. However, Nepal has no established training programmes, and all members of the Association of Nepal’s Occupational Therapists are trained abroad (2019 email from the Association of Nepal’s Occupational Therapists to GJP; unreferenced). Moreover, with only eight occupational therapists servicing the country, there is approximately one occupational therapist per 500,000 persons reported having a form of disability [79]. As total average healthcare spending falls markedly below the global average (Table 3), there is a need to re-allocate public resources to healthcare. Only then can stakeholders facilitate increased training and distribution of specialist clinicians and allied health and social care workers with the specific skills and knowledge to provide high-quality services for older adults. This highlights a need for a multi-modal approach that prioritises investing and reallocating funds to focus on the education of future healthcare providers. Furthermore, to address dire shortages, other strategies may include providing robust incentives to attract people to fields like occupational therapy from other healthcare fields.

Between 48.1% and 72.0% of all health expenditure was out-of-pocket spending, with less than half of all costs financed by the government (Figure 1). Personal spending in Bangladesh accounted for 72.0% of health expenditure, with only 18.2% of healthcare costs covered by the government (Figure 1). This finding indicates significant monetary strain on older adults with few resources to meet the cost of ill-health. Without access to affordable healthcare, vulnerable elderly members of society risk financial catastrophe and poverty due to having to pay for healthcare. This phenomenon was demonstrated by Berman et al. [80], who found that in 2001, out-of-pocket healthcare payments in India and Pakistan propelled 3–25% of the population below the poverty line. The study also showed that, in 2005, the proportion of personal spending on health was 76.1% and 80.9% in India and Pakistan, respectively, highlighting a more than 10% decrease in out-of-pocket expenditure throughout the next decade. By comparison, out-of-pocket expenses in Bangladesh have risen by 15% from 62.6%, whilst Sri Lankan proportions remained relatively unchanged from 46.3% [80]. Without rapid and drastic changes in healthcare financing and improved financial protection for older adults, unregulated out-of-pocket payments constitute a major access barrier to healthcare and undermine country’s progress to achieving UHC. Preventing catastrophic financial expenditure is of particular importance in countries without robust pension systems as retired older adults have no source of revenue. Thus, any financial constraints may directly impact other social determinants of health such as ability to afford quality housing or food, leading to a feedback loop where excessive medical spending further damages health.

Social protection in all study countries is modest, with the Government of Pakistan offering no social pension at all. Pillar Zero (or non-contributory) pensions provide a minimum level of financial protection in Bangladesh and India, costing 0.1 and 0.04 of GDP and covering 34.9% and 17.7% of those eligible, respectively (Table 4). Nepal stands out, with the government spending a slightly more significant proportion of 0.7% of GDP on a social pension above the daily poverty line, and reaching 79.9% of those eligible. However, social security payments for those with dementia are virtually non-existent, apart from India and Nepal, where many people may not be aware of their right to access such funding. For example, although the Nepal’s Government offers several funding provisions to deliver health services to older adults and financial subsidies for selected disease [59], awareness of these schemes is minimal [81,82]. The number of state care homes across each study country is also blatantly disproportionate to need, reflecting family caregiving tradition (Appendix A). However, with the rapid ageing of the population, the government must develop alternative caring arrangements, including specialist dementia provision accessible to older adults, including those in rural or impoverished areas.

### Strengths and weaknesses of the study

It is likely that not all health and socioeconomic resource provisions for older people were identified. The study data were obtained from searches of publicly available literature and supplemented by enquiries to local institutions and personal contacts. Consultation with local experts enabled snowballing of additional relevant reports and data that may not otherwise have been identified. However, the search was conducted in English; therefore, it may have missed results in other languages.

In terms of numbers of various healthcare personnel, it is not known whether the data gathered are representative of human resources available through all sources of healthcare available including public health services, health insurance, non-profit organisations and private medical care. Furthermore, it is uncertain what proportion of each study country's population is accessing government-funded healthcare as, in some countries, only those paying tax are entitled to free healthcare. In these cases, the government average health expenditure may not be accessible to specific older adult populations who need it most. Also, the average spending on herbal and traditional practitioners’ services may not be included in overall average healthcare expenditure but could be a significant source of healthcare provision for older adults in these nations.

Finally, while there are data available on existing or developing legislation and policies, as well as pension schemes and grants concerning provision of care for older adults, further analysis of the implementation and efficacy of these initiatives in the study countries is beyond the scope of this research.

### Possible interpretations and implications for clinicians or policy-makers

As the global movement towards UHC progresses, resource provision gaps for older adults in South Asian countries will present a significant challenge for policy-makers. Above all, the current lack of funding presents a large barrier to reform of existing health infrastructure for older adults. Without rapid increases in health funding, it is inconceivable that health service provision will catch up with the health and social care needs of the rapidly growing older adult population. Additionally, new training programmes for healthcare workers for older adults, including doctors, nurses and allied healthcare professionals, will need steady implementation. This will expand the workforce available for the care of older adults and ensure that patients have access to the necessary specialist health services aimed at older adults. Scaling up recruitment and training in geriatric psychiatry must be a priority to address the growing mental health needs of older adults and address gaps in mental health treatment, prevention and quality of care. Moreover, updating pension schemes and increasing financial support for health and social care will likely lead to improved outcomes for older adults and a reduced illness burden. This is contingent upon services being accessible to the population, including those in rural or impoverished areas.

Primary healthcare is also of paramount importance to older adults. Tham et al. [83] argue that integrated primary healthcare, whereby care is procured collaboratively between primary, secondary and tertiary providers, is essential to look after South Asia’s increasingly ageing and multi-morbid population. Indeed, as populations age and the rates of NCDs increase, policy-makers must emphasise community management of chronic conditions. Wheel et al. [84] support this, stating that primary care reform is crucial to meet the WHO’s agenda in South Asia, which strives to achieve UHC via a community-based, patient-centred and integrated health system. They argue that a lack of funding, lack of dedicated training pathways and a lack of advocacy for primary care at the local and national levels are the key problems in India, Bangladesh, Nepal, Sri Lanka and Pakistan. Sengupta et al. [85] echo this statement, adding that the region’s heterogenous and private-sector-led nature of primary healthcare makes regulation challenging and poses further barriers to reform. Therefore, the priority should be to integrate the various healthcare and social policy reforms with a strengthened primary healthcare system.

### Unanswered questions and future research

Having established what provision there is in countries for older people, future research investigating what data are available on access to care for older adults is required. If data are insufficient, a survey of the level of awareness and uptake of resources in representative populations from South Asian countries, Bangladesh, India, Nepal, Pakistan and Sri Lanka, would be valuable in this regard. Equally, more information provided by study country institutions and personal contacts would be useful where data are not publicly available. Constituting a comprehensive picture of both provision of and access to services which care for older adults in these countries will enable more informed identification of key priorities for clinicians and policy-makers.

The determinants of older adults’ health in South Asian countries extend far beyond healthcare provision and socioeconomic policy. The need to emphasise preventative medicine over curative medicine is highlighted by the fact that the top three causes of death are all non-communicable diseases [86]. Kindig et al. [87] cite that at most anywhere between 10% and 50% of deaths are due to medical causes, with an equal or larger majority caused by behavioural factors and social circumstances. This is supported by Song et al. [88], who highlighted high rates of non-communicable diseases and their risk factors in India, Pakistan, Bangladesh and Sri Lanka through the South Asian Biobank (SAB). Moreover, Adams et al. [89], in their review of grey and published literature on achieving UHC in South Asian cities, claimed that the three key priorities should be urban healthcare governance, action on social determinants of health and affordable healthcare access. Thus, when designing interventions, policy-makers must integrate these multifactorial determinants of health with service provision and existing social policy, rather than viewing different components of healthcare in separate verticals. Given the complexity and breadth of the topic, this review cannot include every determinant of older adult health in South Asian countries, but we hope that the information detailed above is effectively leveraged by policy-makers.

## Conclusion

Inadequate health and socioeconomic resource provision for older adults is an imminent threat to the global ageing population and achieving UHC. This is recognised by the WHO and by country governments. In the study countries, discrepancies in the growing health and social care needs of older adults and available services are indicative of inadequate funding. Although government and non-governmental institutions are working to promote and expand services for older adults, insufficient workforce and government pension and social security schemes present major challenges to existing health and social care systems. There is a need to further recognise geriatrics and geriatric psychiatry as important respective specialities essential to addressing the specific health needs of older adults. This must occur alongside formalising and expanding training programmes to develop a system of healthcare providers for older adults, including a range of allied healthcare professionals. Pension reform and increased financial support for health and social care are also critical to protect an increasingly multimorbid population from catastrophic health expenditure and improve access to those in need. Further investigation of access to health and socioeconomic resources is also essential to guide governing bodies’ future inquiry and, ultimately, priority setting to improve the health of older adults.

## Data Availability

The authors confirm that the data supporting the findings of this study are available within the article and its supplementary materials.

## Paper context

The fast demographic transition in LMICs requires a significant increase in resources to meet the ageing population’s health and social care needs. This review article demonstrates that, in Bangladesh, India, Nepal, Pakistan and Sri Lanka, the numbers of health professionals with geriatric expertise, health funding and pension and social security schemes are grossly inadequate. To achieve universal health coverage, governing bodies must scale up care for older adult training programmes and increase social protection for those in need.

## Appendix A. Social protection programmes for the elderly

**Table:**

	Bangladesh	India	Nepal	Pakistan	Sri Lanka
**Retirement age (years)**	Public sector (freedom fighters): 60, the government has implemented a court directive to lengthen service by 1 yearPublic sector (non-freedom fighters): 59^1^	Public sector: 60,^2^ raised to 65 years for central government doctors^3^ Private sector: 58^2^	Public sector: 58, but deferred in certain instances Health professionals and Parliament workers: 60 Teachers: 63 Court workers: 65^4^	60 (men), 55 (women), with at least 15 years of contributions; miners can claim the pension earlier under certain conditions^5^ Government has proposed for retirement age to be increased to 63^6^	55^7^
**Old age social pension**	*Old Age Allowance* Targeting approach: income-tested Eligibility criteria: age ≥65 (men) and ≥62 (women) with an annual income of <10,000 takas ($118), not receiving any other government or nongovernment allowance, only one member of a family can receive the pension Monthly benefit: 500 takas ($6) (paid every 3 months)^5,8^ Government plans to introduce a universal pension scheme for private sector jobholders^9^	*Indira Gandhi National Old Age Pension Scheme* Targeting approach: income-tested Eligibility criteria: age ≥60, with an annual income not exceeding a certain limit, which may vary across states Monthly benefit: 200 rupees if age 60–79500 rupees if aged ≥80; additional amounts may be paid and vary by state^5,10^ *Indira Gandhi National Widow Pension Scheme*Targeting approach: income-tested Eligibility criteria: widows aged ≥40, with annual income not exceeding a certain limit, which may vary by stateMonthly benefit: 300 rupees^5,10^	*Old Age Allowance* Targeting approach: pensions-tested Eligibility Criteria: age ≥65 (≥60 for Dalits, single and divorced women, and residents of Karnali Zone)Monthly benefit: 2000 rupees; 1,000 for members of the Rautes, Chepang, and certain other ethnic groups (paid every four months)^5,11^	Social insurance system^5,12^	*Senior Citizen’s Allowance for Strengthen Elderly* Targeting approach: income-testedEligibility criteria: age ≥70 with income below a certain thresholdMonthly benefit: 2000 Sri Lankan rupees^13^
**Social protection for people with dementia**	No financial benefits/social protection for dementia carers^14^	*Indira Gandhi National Disability Pension Scheme* Targeting approach: income-tested Eligibility criteria: aged ≥18, with an assessed degree of disability ≥80% and annual income not exceeding a certain limit, which may vary across statesMonthly benefit: 300 rupees (central contribution), additional amounts may be paid and vary by state^5^	Department of Health Services provides 100,000 Nepalese rupees to patients diagnosed with Alzheimer’s disease, to cover the cost of healthcare from four enlisted hospitals (National Academy of Medical Sciences, Tribhuvan University Teaching Hospital and Patan Academy of Health Sciences in Kathmandu Valley and BP Koirala Institute of Health Sciences in Dharan)^20^	No government policy for people living with dementia^21^	Not available
		*Integrated Programme for Older Persons Scheme* Provides funding for up to 90% of the project costs to eligible agencies for establishing and maintaining old-age homes, day care centres, mobile medicare units and delivering non-institutional services to older persons–includes the running of day care centres for patients with dementia^15^ *Assistance for construction of old-age homes for older persons Scheme* Provides funding for construction of old-age homes–Scheme is presently under formulation^15^			
		*Rashtriya Vayoshri Yojana* Provides physical aids and assisted-living devices to senior citizens belonging to the below the poverty line (BPL) category^16^ *Pradhan Mantri Jan Arogya Yojana* Provides coverage of up to 500,000 rupees per family per year for secondary and tertiary hospitalisation to over 107.4 million eligible poor and vulnerable families (approximately 500 million beneficiaries)^17^			
		*Welfare of Parents and Senior Citizens Act 2007* Mandates needs-based maintenance (food, clothing, residence, medical attendance and treatment) for parents/grandparents from their children to protect the life and property of senior citizens^15^ *Income Tax Act 1961*Section 80 U: deduction can be claimed by an individual if they are certified by the medical authority to be a person with disability^18^ Section 80DDB: deduction can be claimed by an individual for themselves or a dependant relative, for expenditures incurred for medical treatment of specified diseases,^18^ including dementia^19^			
**State care homes**	Community-based services for dementia are available, but in the capital and main cities only^14^ Six aged care homes (Santi Nibas) in six divisions^22^	Six full-time residential care facilities and around ten day-care services exclusively for people with dementia^23^ Welfare of Parents and Senior Citizens Act 2007 contains enabling provisions for setting up of old-age homes for providing maintenance to senior citizens^15^	70 old-age homes (OAHs), of which 11 get government grants^24^ One government-run residential facility (Pashupati Bridrashram), with the capacity for 230 older adults^24,25^	Very few old-age homes located in Karachi, run by charities, though none specifically for people with dementia^26^ Families are more likely to accept home help than send persons with dementia to a nursing home^21^ No tradition of old-age homes, reliance is on older adults co-residing with family^27^	Most residential care homes are run by local, often faith-based, voluntary social service organisations: two-thirds have 30 places or fewer, 18% have more than 50 places and there are a growing number of private fee-paying long-term care residential homes, most in or near Colombo, caring for 10–30 people^28^

^1^Tribune Desk. Public sector freedom fighters’ retirement age extended. Dhaka Tribune [Internet]. 11 April 2018 [cited 12 June 2019]. Available from: https://www.dhakatribune.com/bangladesh/2018/04/11/public-sector-freedom-fighters-retirement-age-extended.

^2^TNN. At 58, retirement age in India is one of the lowest worldwide. The Times of India [Internet]. 25 April 2018 [cited 12 June 2019]. Available from: http://timesofindia.indiatimes.com/articleshow/63905499.cms?utm_source=contentofinterest&utm_medium=text&utm_campaign=cppst.

^3^Express Web Desk. Retirement age of central govt doctors raised to 65 years. The Indian Express [Internet]. 27 September 2017 [cited 12 June 2019]. Available from: https://indianexpress.com/article/india/retirement-age-of-central-govt-doctors-to-be-increased-to-65-years-4863882/.

^4^Panday J. Government to revise retirement age for everyone on its payrolls to 60 years. The Himalayan Times [Internet]. 1 August 2018 [cited 12 June 2019]. Available from: https://thehimalayantimes.com/nepal/government-to-revise-retirement-age-for-everyone-on-its-payrolls-to-60-years/.

^5^The USA Social Security Administration. Social Security Programs Throughout the World: Asia and the Pacific, 2018. Washington, D.C.: Social Security Administration; 2019. (SSA Publication No. 13.11802).

^6^Ashfaq M. Govt to increase retirement age by three years. DAWN [Internet]. 12 December 2018 [cited 12 June 2019]. Available from: https://www.dawn.com/news/1450879.

^7^Salary.lk. Social Security and Pensions in Sri Lanka [Internet]. [cited 12 June 2019]. Available from: https://salary.lk/labour-law/social-security/social-security-and-pensions-1.

^8^Pension Watch. Bangladesh [Internet]. [cited 6 April 2020]. Available from: http://www.pension-watch.net/country-fact-file/bangladesh.

^9^Senior Correspondent. Bangladesh plans to embrace universal pension era. bdnews24com [Internet]. 8 June 2018 [cited 12 June 2019]. Available from: https://bdnews24.com/economy/2018/06/08/bangladesh-plans-to-embrace-universal-pension-era.

^10^Pension Watch. India [Internet]. [cited 6 April 2020]. Available from: http://www.pension-watch.net/country-fact-file/india.

^11^Pension Watch. Nepal [Internet]. [cited 6 April 2020]. Available from: http://www.pension-watch.net/country-fact-file/nepal.

^12^Pension Watch. Pakistan [Internet]. [cited 6 April 2020]. Available from: http://www.pension-watch.net/country-fact-file/pakistan.

^13^Pension Watch. Sri Lanka [Internet]. [cited 6 April 2020]. Available from: http://www.pension-watch.net/country-data/sri-lanka/.

^14^World Health Organization. Global Dementia Observatory (GDO) Provisional Country Profile 2017: Bangladesh [Internet]. [cited 12 June 2019]. Available from: https://www.who.int/mental_health/neurology/dementia/bangladesh_GDO_profile.pdf.

^15^Ministry of Social Justice and Empowerment. Senior Citizen Division [Internet]. [cited 5 February 2021]. Available from: http://socialjustice.nic.in/Home/SiteSearch?Search=senior%20citizen%20division.

^16^National Portal of India. Rashtriya Vayoshri Yojana [Internet]. [cited 14 May 2020]. Available from: https://www.india.gov.in/spotlight/rashtriya-vayoshri-yojana.

^17^National Health Authority. Ayushman Bharat Pradhan Mantri Jan Arogya Yojana [Internet]. [cited 5 February 2021]. Available from: https://www.pmjay.gov.in/about/pmjay.

^18^Income Tax Department. Government of India. Deductions [Internet]. [cited 11 April 2020]. Available from: https://www.incometaxindia.gov.in/_layouts/15/dit/Pages/viewer.aspx?path=https://www.incometaxindia.gov.in/Charts%20%20Tables/Deductions.htm&grp=&searchFilter=&k=&IsDlg=0.

^19^Income Tax Department. Government of India. Specified diseases and ailments for the purpose of deduction under section 80DDB [Internet]. [cited 11 April 2020]. Available from: https://incometaxindia.gov.in/Rules/Income-Tax%20Rules/103120000000007232.htm.

^20^Koirala S. Alzheimer’s Disease in Nepal. Nepal: HelpAge International; 2016. http://ageingnepal.org/wp-content/uploads/2016/09/Alzheimers-disease-in-Nepal_final_sharad.pdf.

^21^Zaidi A, Willis R, Farina N, Balouch S, Jafri H, Ahmed I Understanding, Beliefs and Treatment of Dementia in Pakistan: Final Report. Age International, Age UK & HelpAge International; 2019. https://eprints.soton.ac.uk/429832/1/DiP_Final_Report_31_January_2019.pdf.

^22^Hossain I, Akhtar T, Uddin T. The Elderly Care Services and their Current Situation in Bangladesh: An Understanding from Theoretical Perspective. J Med Sci. 2006;6(2):131–8.

^23^Shaji KS, Jotheeswaran AT, Girish N, Bharath S, Dias A, Pattabiraman M The Dementia India Report 2010: Prevalence, impact, costs and services for dementia. New Delhi: Alzheimer’s and Related Disorders Society of India; 2010. (ISBN978819203410 2).

^24^Shrestha L. Residential Care Home for Elderly People in Nepal: Geriatric health in Nepal [Internet]. [cited 12 June 2019]. Available from: https://www.ifa-fiv.org/wp-content/uploads/2013/03/IFA-presentation-2014.pdf.

^25^Shrestha L. Geriatric Health in Nepal: Concerns and Experience. Nepal Med Coll J. 2012;15(2):144–8.

^26^2020 email from M Khan to NRM; unreferenced.

^27^Majid H, Memon S. Elderly care in Pakistan. DAWN [Internet]. 19 February 2018 [cited 12 June 2019]. Available from: https://www.dawn.com/news/1390240.

^28^United Nations Economic and Social Commission for Asia and the Pacific. SDD-SPPS Project Working Papers Series: Long-term Care for Older Persons in Asia and the Pacific: Long-term Care for Older Persons in Sri Lanka. Bangkok: United Nations Economic and Social Commission for Asia and the Pacific; 2016.

## References

[1] United Nations Department of Economic and Social Affairs. World population prospects: the 2017 revision, key findings and advance tables. New York: United Nations; 2017. (Working Paper No. ESA/P/WP/248).

[2] World Health Organization. Ageing and health [Internet]. 2018. [cited 2019 May 31]. Available from: https://www.who.int/news-room/fact-sheets/detail/ageing-and-health

[3] Abegunde DO, Mathers CD, Adam T, et al. The burden and costs of chronic diseases in low-income and middle-income countries. Lancet. 2007;370(9603):1929–16. 18063029 10.1016/S0140-6736(07)61696-1

[4] Gulliford M, Ravindrarajah R. Frailty: from clinical syndrome to epidemiological construct? Lancet Public Health. 2018;3(7):305–306. 10.1016/S2468-2667(18)30112-9 29908858

[5] Chomik R, Piggott J. Population ageing and social security in Asia. Asian Econ. 2015;10(2):199–222.

[6] Afshar S, Roderick PJ, Kowal P, et al. Multimorbidity and the inequalities of global ageing: a cross-sectional study of 28 countries using the World Health Surveys. BMC Public Health. 2015;15:776. 26268536 10.1186/s12889-015-2008-7 PMC4534141

[7] Arokiasamy P, Uttamacharya U, Jain K, et al. The impact of multimorbidity on adult physical and mental health in low-and middle-income countries: what does the study on global ageing and adult health (SAGE) reveal? BMC Med. 2015;13:178. 26239481 10.1186/s12916-015-0402-8 PMC4524360

[8] Jotheeswaran AT, Bryce R, Prina M, et al. Frailty and the prediction of dependence and mortality in low-and middle-income countries: a 10/66 population-based cohort study. BMC Med. 2015;13:138. 26063168 10.1186/s12916-015-0378-4 PMC4481121

[9] World Health Organization. Ageing and life-course: healthy ageing and the sustainable development goals [Internet]. [cited 2020 Apr 10]. Available from: https://www.who.int/ageing/sdgs/en/.

[10] Whiteford HA, Degenhardt L, Rehm J, et al. Global burden of disease attributable to mental and substance use disorders: findings from the global burden of disease study 2010. Lancet. 2013;382(9904):1575–1586. 23993280 10.1016/S0140-6736(13)61611-6

[11] Steel Z, Marnane C, Iranpour C, et al. The global prevalence of common mental disorders: a systematic review and meta-analysis 1980–2013. Int J Epidemiol. 2014;43(2):476–493. 24648481 10.1093/ije/dyu038 PMC3997379

[12] World Health Organization. Depression and other common mental disorders: global health estimates. Geneva: World Health Organization; 2017. (WHO Reference No. WHO/MSD/MER/2017.2).

[13] World Health Organization. Mental Health Gap Action Programme (mhGAP): scaling up care for mental, neurological, and substance use disorders. Geneva: World Health Organization; 2008. 26290926

[14] Chisholm D, Sweeny K, Sheehan P, et al. Scaling-up treatment of depression and anxiety: a global return on investment analysis. Lancet Psychiatry. 2016;3(5):415–424. 27083119 10.1016/S2215-0366(16)30024-4

[15] Patel V, Xiao S, Chen H, et al. The magnitude of and health system responses to the mental health treatment gap in adults in India and China. Lancet. 2016;388(10063):3074–3084. 27209149 10.1016/S0140-6736(16)00160-4

[16] World Health Organization, World Organization of Family Doctors. Integrating mental health into primary care: a global perspective. Geneva; London: World Health Organization, World Organization of Family Doctors; 2008. (WHO Reference No. WM 140 2008IN).

[17] World Health Organization. Mental health action plan 2013-2020. Geneva: World Health Organization; 2013.

[18] World Health Organization. Active ageing: towards age-friendly primary health care. Geneva: World Health Organization; 2004.

[19] World Health Organization. Age-friendly primary health care centres toolkit. Geneva: World Health Organization; 2008.

[20] Lund C, Breen A, Flisher AJ, et al. Poverty and common mental disorders in low and middle income countries: a systematic review. Soc Sci Med. 2010;71(3):517–528. 20621748 10.1016/j.socscimed.2010.04.027 PMC4991761

[21] Albanese E, Liu Z, Acosta D, et al. Equity in the delivery of community healthcare to older people: findings from 10/66 Dementia research group cross-sectional surveys in Latin America, China, India and Nigeria. BMC Health Serv Res. 2011;11:153. 21711546 10.1186/1472-6963-11-153 PMC3146820

[22] World Bank. World Bank Open Data [Internet]. [cited 2020 Apr 9]. Available from: https://data.worldbank.org/.

[23] Sharma SG Geriatric care in India still in its infancy. Hindustan Times [Internet]. 2015 Aug 20 [cited 2019 Jul 10]. Available from: https://www.hindustantimes.com/health-and-fitness/geriatric-care-in-india-still-in-its-infancy/story-SsPNcQFSmgPrWwVv8q11MP.html.

[24] Khan S, Itrat M. Current issues in geriatric health care in India-a review. J Community Med Health Care. 2016;1(1):1003.

[25] Khan AT, Toor RH, Amjad Q. Assessment and management of geriatric care in Pakistan. J Gerontol Geriatr Res. 2018;7(5):488.

[26] World Health Organization. Global health observatory data repository: human resources data by country [Internet]. 2019. [cited 2019 Jun 7]. Available from: http://apps.who.int/gho/data/node.main.MHHR?lang=en.

[27] World Health Organization. Global Dementia Observatory (GDO) Provisional Country Profile 2017: Bangladesh [Internet]. 2017. [cited 2019 Jun 12]. Available from: https://www.who.int/mental_health/neurology/dementia/bangladesh_GDO_profile.pdf.

[28] Indian Association for Geriatric Mental Health. Indian Association for geriatric mental health: membership directory [Internet]. 2019. [cited 2019 Nov 29]. Available from: https://iagmh.org/register-list/uploads/docs/IAGMH Updated Membership Directory.pdf.

[29] World Confederation for Physical Therapy. Bangladesh WCPT Country Profile 2019 [Internet]. 2019. [cited 2020 Apr 11]. Available from: https://www.wcpt.org/sites/wcpt.org/files/files/cds/reports/2019/150013.pdf

[30] The Indian Association of Physiotherapists. IAP At a Glance [Internet]. 2020. [cited 2020 Apr 24]. Available from: http://www.physiotherapyindia.org/about/index.1.html.

[31] World Confederation for Physical Therapy. Nepal WCPT Country Profile 2019 [Internet]. 2019. [cited 2020 Apr 11]. Available from: https://www.wcpt.org/sites/wcpt.org/files/files/cds/reports/2019/150128.pdf

[32] World Confederation for Physical Therapy. WCPT Country Profile December 2017: Pakistan [Internet]. 2017. [cited 2020 Apr 11]. Available from: https://www.wcpt.org/sites/wcpt.org/files/files/cds/reports/2017/150136.pdf.

[33] World Confederation for Physical Therapy. Sri Lanka society of physiotherapy [Internet]. 2020. [cited 2020 Jun 20]. Available from: https://www.wcpt.org/node/26760.

[34] Bangladesh Occupational Therapy Association. Executive members [Internet]. [cited 2019 Jul 10]. Available from: https://www.bota.org.bd/members.php?typeID=2.

[35] Bangladesh Occupational Therapy Association. Professional members [Internet]. [cited 2019 Jul 10]. Available from: https://www.bota.org.bd/members.php?typeID=4.

[36] Canadian Physiotherapy Association. Country profile: Pakistan [Internet]. [cited 2019 Jun 7]. Available from: https://physiotherapy.ca/country-profile-pakistan.

[37] Indian Speech-Language and Hearing Association. Welcome to ISHA [Internet]. [cited 2019 Jun 7]. Available from: https://www.ishaindia.org.in/.

[38] Director Ziauddin College of Speech Language Therapy. Speech language therapy in Pakistan. DAWN [Internet]. 2010 Aug 22; [cited 2019 Jun 7]. Available from: https://www.dawn.com/news/880784/speech-language-therapy-in-pakistan.

[39] de Silva N, Shadden BB, Hagstrom F, et al. Developing the speech-language therapy profession in Sri Lanka: international collaborations. Oral presentation presented at: American Speech-Language-Hearing Association Convention; 2013 Nov 14–16; Chicago, IL.

[40] World Health Organization. Global health observatory data: psychiatrists and nurses (per 100 000 population) [Internet]. 2019. [cited 2019 Jul 17]. Available from: https://www.who.int/gho/mental_health/human_resources/psychiatrists_nurses/en/.

[41] Medical Council of India. College and course search [Internet]. [cited 2020 May 14]. Available from: https://www.mciindia.org/CMS/information-desk/college-and-course-search.

[42] Bangladesh Health Professions Institute. Department of speech & language therapy overview [Internet]. [cited 2019 Jul 10]. Available from: https://www.bhpi.edu.bd/department/overview/.

[43] Indian Speech-Language and Hearing Association. The professional [Internet]. [cited 2019 Jul 10]. Available from: https://www.ishaindia.org.in/professional.html.

[44] doctorsbd.com. Doctor search [Internet]. [cited 2019 Jul 10]. Available from: https://www.doctorsbd.com/.

[45] Barikdar A, Ahmed T, Lasker SP. The situation of the elderly in Bangladesh. Bangladesh J Bioeth. 2016;7(1):27–36.

[46] Senior Correspondent. DMCH special unit for elderly care. Bdnews24com [Internet]. 2014 Oct 1; [cited 2019 Jul 10]. Available from: https://bdnews24.com/health/2014/10/01/dmch-special-unit-for-elderly-care.

[47] Kabir R, Kabir M, Uddin MSG, et al. Elderly population growth in Bangladesh: preparedness in public and private sectors. IOSR-JHSS. 2016;21(8):58–73.

[48] International Federation on Ageing. Bangladesh Association for the Aged and Institute of Geriatric Medicine (BAAIGM) [Internet]. [cited 2019 Jun 7]. Available from: https://www.ifa-fiv.org/partner-profiles/bangladesh-association-for-the-aged-and-institute-of-geriatric-medecine-baaigm/.

[49] Bangladesh Association for the Aged and Institute of Geriatric Medicine. Research and publication [Internet]. [cited 2020 Apr 8]. Available from: https://baaigm.org.bd/research-and-publication/.

[50] Subarta Trust. Projects [Internet]. [cited 2019 Dec 18]. Available from: http://subarta.org/projects/.

[51] Sir William Beveridge Foundation. Health & social care bangladesh [Internet]. [cited 2019 Dec 18]. Available from: http://www.beveridgefoundation.org/projects-international/health-social-care-bangladesh/.

[52] Evans JM, Kiran PR, Bhattacharyya OK. Activating the knowledge-to-action cycle for geriatric care in India. Health Res Policy Syst. 2011;9:42. 22136552 10.1186/1478-4505-9-42 PMC3254590

[53] Verma R, Khanna P. National program of health-care for the elderly in India: a hope for healthy ageing. Int J Prev Med. 2013;4(10):1103–1107. 24319548 PMC3843295

[54] Ministry of Health and Family Welfare. National Programme for Health Care of the Elderly (NPHCE) [Internet]. [cited 2020 May 14]. Available from: https://main.mohfw.gov.in/major-programmes/other-national-health-programmes/national-programme-health-care-elderlynphce.

[55] Indian Academy of Geriatrics. Indian Academy of Geriatrics [Internet]. [cited 2019 Dec 4]. Available from: http://www.indianacademyofgeriatrics.com/index.html.

[56] Geriatric Society of India. Welcome to Geriatric Society of India [Internet]. [cited 2019 Dec 4]. Available from: https://www.geriatricindia.com/index.html.

[57] King George’s Medical University. Geriatric Mental Health [Internet]. [cited 2019 Dec 4]. Available from: http://www.kgmu.org/department_details.php?dept_id=14&dept_type=2.

[58] Nepalese Society of Gerontology and Geriatrics (NSGG). Aim [Internet]. [cited 2019 Jun 7]. Available from: http://gerontonepal.org/aim/.

[59] Shrestha L. Geriatric Health in Nepal: concerns and Experience. Nepal Med Coll J. 2012;15(2):144–148. 24696938

[60] World Health Organization. Mental Health Atlas-2011 country profiles: Pakistan [Internet]. 2010. [cited 2020 Apr 22]. Available from: https://www.who.int/mental_health/evidence/atlas/profiles/pak_mh_profile.pdf?ua=1.

[61] Samaraweera D, Maduwage S. Meeting the current and future health-care needs of Sri Lanka’s ageing population. WHO South-East Asia J Public Health. 2016;5(2):96–101. 28607235 10.4103/2224-3151.206259

[62] Postgraduate Institute of Medicine University of Colombo. Courses [Internet]. [cited 2021 Feb 5]. Available from: https://pgim.cmb.ac.lk/index.php/courses/.

[63] Lanka Doctor. Find a Doctor [Internet]. [cited 2019 Jul 10]. Available from: https://lankadoctor.com/index.php.

[64] Sri Lanka Association of Geriatric Medicine. Who we are [Internet]. [cited 2021 Mar 31]. Available from: https://www.slagm.lk/about-slagm/.

[65] National Institute of Mental Health Sri Lanka. Psycho-Geriatric unit [Internet]. [cited 2020 Apr 3]. Available from: http://nimh.health.gov.lk/en/psycho-geriatric-unit/.

[66] National Institute of Mental Health Sri Lanka. Our team members [Internet]. [cited 2020 Apr 3]. Available from: http://nimh.health.gov.lk/en/our-team-members/.

[67] World Health Organization. The World Health Report: health systems financing: the path to universal coverage. Geneva: World Health Organization; 2010. 10.2471/BLT.10.078741 PMC2878164 20539847

[68] Institute for Health Metrics and Evaluation. Financing Global Health Visualization: viz Hub [Internet]. [cited 2020 May 17]. Available from: http://vizhub.healthdata.org/fgh/.

[69] Pension Watch. Pakistan [Internet]. [cited 2020 Apr 6]. Available from: http://www.pension-watch.net/country-fact-file/pakistan.

[70] World Bank. The World Bank pension conceptual framework. Washington, D.C: World Bank; 2008. (No. 45728)

[71] Pension Watch. HelpAge International: Social pensions database [Internet]. [cited 2019 Jun 12]. Available from: http://www.pension-watch.net/social-pensions-database/social-pensions-database–/.

[72] Organisation for Economic Co-operation and Development. Pensions at a glance 2017: OECD and G20 indicators. Paris: OECD Publishing; 2017.

[73] Organisation for Economic Co-operation and Development. OECD family database CO1.2: life expectancy at birth [Internet]. [cited 2021 Feb 5]. Available from: https://www.oecd.org/els/family/CO_1_2_Life_expectancy_at_birth.pdf.

[74] Patel V, Saxena S, Lund C, et al. The Lancet Commission on global mental health and sustainable development. Lancet. 2018;392(10157):1553–1598. 30314863 10.1016/S0140-6736(18)31612-X

[75] Lodha P, De Sousa A. Geriatric mental health: the challenges for India. J Geriatr Ment Health. 2018;5(1):16–29.

[76] Shaji KS, Jotheeswaran AT, Girish N, et al. The Dementia India Report 2010: prevalence, impact, costs and services for dementia. New Delhi: Alzheimer’s and Related Disorders Society of India; 2010.

[77] Welsh TJ, Gordon AL, Gladman JR. Comprehensive geriatric assessment – a guide for the non-specialist. Int J Clin Pract. 2014;68(3):290–293. 24118661 10.1111/ijcp.12313 PMC4282277

[78] All India Occupational Therapists’ Association. All India occupational therapists’ association: summary of discussions [Internet]. [cited 2020 Jul 10]. Available from: http://aiota.org/temp/site/images/Download.pdf.

[79] Unity in Health. Planting the seeds for Occupational Therapy training in Nepal [Internet]. [cited 2020 Apr 8]. Available from: https://unityinhealth.org/planting-the-seeds-for-occupational-therapy-training-in-nepal/.

[80] Berman P, Ahuja R, Bhandari L. The impoverishing effect of healthcare payments in India: new methodology and findings. Econ Political Wkly. 2010;45(16):65–71.

[81] Bisht PS, Pathak RS, Subedi G, et al. Health and social care needs assessment of elderly: the context of piloting service developments and care of elderly in Pharping, Kathmandu, Nepal. Kathmandu: United Nations Population Fund (UNFPA); 2012.

[82] Acharya S, Ghimire S, Jeffers EM, et al. Health care utilization and health care expenditure of Nepali older adults. Front Public Health. 2019;7:24. 30828573 10.3389/fpubh.2019.00024 PMC6384236

[83] Tham TY, Tran TL, Prueksaritanond S, et al. Integrated health care systems in Asia: an urgent necessity. Clin Interv Aging. 2018;13:2527–2538. 30587945 10.2147/CIA.S185048 PMC6298881

[84] van Weel C, Kassai R, Qidwai W, et al. Primary healthcare policy implementation in South Asia. BMJ Glob Health. 2016;1(2):e000057. 10.1136/bmjgh-2016-000057 PMC5321321 28588938

[85] Sengupta A, Zaidi S, Sundararaman T, et al. Tackling the primary care access challenge in South Asia. BMJ. 2018;363:k4878. 30498073 10.1136/bmj.k4878

[86] World Health Organization. The top 10 causes of death [Internet]. [cited 2021 Dec 13]. Available from: https://www.who.int/news-room/fact-sheets/detail/the-top-10-causes-of-death.

[87] Kindig DA, Asada Y, Booske B. A population health framework for setting national and state health goals. JAMA. 2008;299(17):2081–2083. 18460667 10.1001/jama.299.17.2081

[88] Song P, Gupta A, Goon IY, et al. Data resource profile: understanding the patterns and determinants of health in South Asians—the South Asia Biobank. Int J Epidemiol. 2021;50(3):717–8e. 34143882 10.1093/ije/dyab029 PMC8271208

[89] Adams AM, Nambiar D, Siddiqi S, et al. Advancing universal health coverage in South Asian cities: a framework. BMJ. 2018;363:k4905. 30498010 10.1136/bmj.k4905 PMC7115914

